# Control failure risk, resistance and enzymatic activity of neurotoxic insecticides in Brazilian populations of *Leucoptera coffeella* (Lepidoptera: Lyonetiidae)

**DOI:** 10.1002/ps.70677

**Published:** 2026-02-22

**Authors:** Daianna P. Costa, Carlos G. da Cruz, Ryan F. S. e Silva, Liliane E. Visôtto, Jesús E. Gomez, Marisol Giraldo‐Jaramillo, Flávio L. Fernandes

**Affiliations:** ^1^ Instituto de Ciências Agrárias, Universidade Federal de Viçosa, Rio Paranaíba Rio Paranaíba Brazil; ^2^ Departament Agroambiental Science Puerto Rico University‐Mayagüez San Juan Puerto Rico; ^3^ Centro Nacional de Investigaciones de Café/Cenicafé Caldas Colombia

**Keywords:** *Coffea* spp., coffee leaf miner, insecticides, metabolic enzymes

## Abstract

**Background:**

*Leucoptera coffeella* is a key pest of coffee crops in Brazil, causing significant damage by mining coffee leaves. Reports of reduced effectiveness of chemical control measures highlight the need to investigate potential resistance mechanisms. This study aimed to evaluate the risk of control failure, resistance levels and enzymatic activity in Brazilian field populations of *L. coffeella* exposed to neurotoxic insecticides.

**Results:**

A total of 35 field populations of *L. coffeella* were collected and tested in the laboratory using filter paper discs treated with insecticide solutions. After 48 h of exposure, mortality rates, median and 95% lethal concentrations (LC_50_ and LC_95_), and median resistance ratios (RR_50_) were determined. Control failure risk was identified in 34.3% and 62.9% of populations for abamectin, chlorpyrifos, deltamethrin and profenophos. Five populations exhibited low to very high resistance to abamectin (RR_50_ = 5.5–794), and two populations showed very high resistance to chlorpyrifos (RR_50_ = 142 and 727) and deltamethrin (RR_50_ = 1281 and 1656). Enzymatic assays revealed lower acetylcholinesterase activity in Rio Paranaíba II and higher glutathione *S*‐transferase activity in Rio Paranaíba I, followed by Carmo do Paranaíba populations.

**Conclusion:**

Our findings demonstrate widespread resistance and altered enzymatic activity in *L. coffeella* populations across Brazilian coffee‐growing regions. These results highlight the urgent need to incorporate resistance monitoring and alternative control strategies into integrated pest management programs to ensure sustainable coffee production. © 2026 The Author(s). *Pest Management Science* published by John Wiley & Sons Ltd on behalf of Society of Chemical Industry.

## Graphical abstract

Bioassays of 36 *Leucoptera coffeella* populations revealed resistance to neurotoxic insecticides, control failures of ≤62.9% and enzyme activity variation, with particularly high GST and low AChE levels.

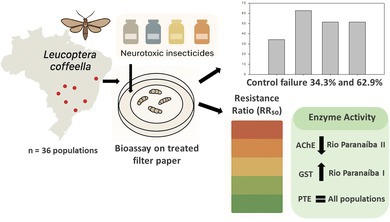

## 1 INTRODUCTION

One of the challenges of modern agriculture and farm management is reducing the costs associated with the production process.^1^ In the production process, diseases, weed, and pests must be controlled to avoid losses. In an attempt to control pests, insecticides are frequently used, which increases production costs.^2^ These costs can be even higher if issues with pest resistance arise.^3^

Insecticide resistance was first reported in 1914 by A. L. Melander.^4^ It can be defined as the ability of an insect to withstand insecticide doses that would be lethal to most members of a susceptible population. When the proportion of resistant individuals within an insect population increases, it can impair and/or reduce the effectiveness of the insecticide intervention.^5^, ^6^ Resistance and control failure have been documented in various agricultural pest insects, demonstrating that these phenomena are widely observed worldwide.^7^, ^8^, ^9^ Although resistance should not be confused with control failure, both phenomena may be associated. Control failure owing to insecticide resistance occurs when the efficacy of an insecticidal formulation, used at its prescribed rate, falls significantly below its expected control level.^10^

In general, insecticide resistance is caused by behavioral modifications, reduced cuticular penetration, modifications to insecticide target sites and increased metabolic enzyme activity. The relationship between insecticide resistance and insect enzyme activity has been widely reported in numerous insect orders^11^, ^12^ and involves several different types of reactions. These reactions are catalyzed by a multi‐enzymatic system, including glutathione *S*‐transferase (GST), cytochrome P450‐dependent monooxygenases, and esterases, which catalyze the detoxification of exogenous molecules.^13^, ^14^, ^15^ These enzymes reduce the persistence of insecticides within insects by catalyzing their detoxification, contributing to resistance.^16^ Esterases are frequently implicated in insect resistance to organophosphates and pyrethroids.^17^, ^18^ Insensitivity of a target site to organophosphates and pyrethroids is usually related to acetylcholinesterase activity (AChE).^19^ GST activity has been associated with resistance to all main classes of insecticides, which together can provide multilayered chemical protection.^14^ In this context, the coffee leaf miner, *Leucoptera coffeella* (Guérin‐Méneville) (Lepidoptera: Lyonetiidae), represents an important model for investigating insecticide resistance mechanisms.

*Leucoptera coffeella* is monophagous and native to Africa, but has become a pest of *Coffea arabica* and *C. canephora* (L.) in several countries.^20^ This pest has a variable lifecycle and high destructive capacity in coffee crops. In the state of Minas Gerais, Brazil's largest coffee producer, the lifecycle can vary from 11 to 46 days, depending primarily on ambient temperature.^21^, ^22^ Adult females lay their eggs on the adaxial surface of leaves. Immediately after eclosion, the 1^st^‐instar larvae penetrate leaves and begin mining near the adaxial surface. After 8–10 days, the larvae exit the leaves, move to the leaf's abaxial surface and mature into the pupae. These attacks reduce photosynthesis, causing leaves to fall and resulting in losses as high as 90%.^23^

In Brazil, there are currently 210 commercial insecticide formulations for coffee leaf miners, containing 28 different active ingredients that are present either singly or in combination.^24^ Despite the availability of various active ingredients that enable pesticide rotation, documented usage profiles show that Brazilian farmers frequently apply high rates of the same insecticide formulations for coffee leaf miner control.^25^ This practice, widely recognized in the literature, increases the risk of selecting resistant pest populations.^26^ Nevertheless, research on insecticide resistance in *L. coffeella* has only been carried out with organophosphorus.^27^, ^28^, ^29^, ^30^ and diamide^25^ insecticides. The most common insecticides are neurotoxins that tend to result in higher rates of resistance.^31^ Studies on insecticide resistance and control failure for the coffee leaf miner have been limited. Despite the importance of *L. coffeella* pesticide resistance, little is known about the underlying mechanisms.

Therefore, we assessed the (a) risk of control failures, (b) resistance levels, and (c) enzymatic activity in Brazilian populations of *L. coffeella* to four neurotoxic insecticides.

## 2 MATERIALS AND METHODS

### 2.1 Insecticides

Four insecticides were used in this study. Abamectin 18 EC and profenophos 550 EC were purchased from Syngenta Ltda (Ribeirão Preto, SP, Brazil), and chlorpyrifos 480 EC and deltamethrin 25 EC were purchased from Du Pont – Brazil S.A (Barueri, SP, Brazil) and Bayer S.A (Belford Roxo, RJ, Brazil), respectively. Bovine serum albumin (BSA), acetylthiocholine iodide (ATC), 5′,5′‐dithiobis 2‐nitrobenzoic acid (DTNB), glutathione, 3,4‐dichloro‐2,4‐nitrobenzene (DCNB), paraoxon and ethylenediaminetetraacetic acid (EDTA) were purchased from Sigma‐Aldrich Química Brazil Ltda (São Paulo, SP, Brazil).

### 2.2 Population

**Figure 1:**
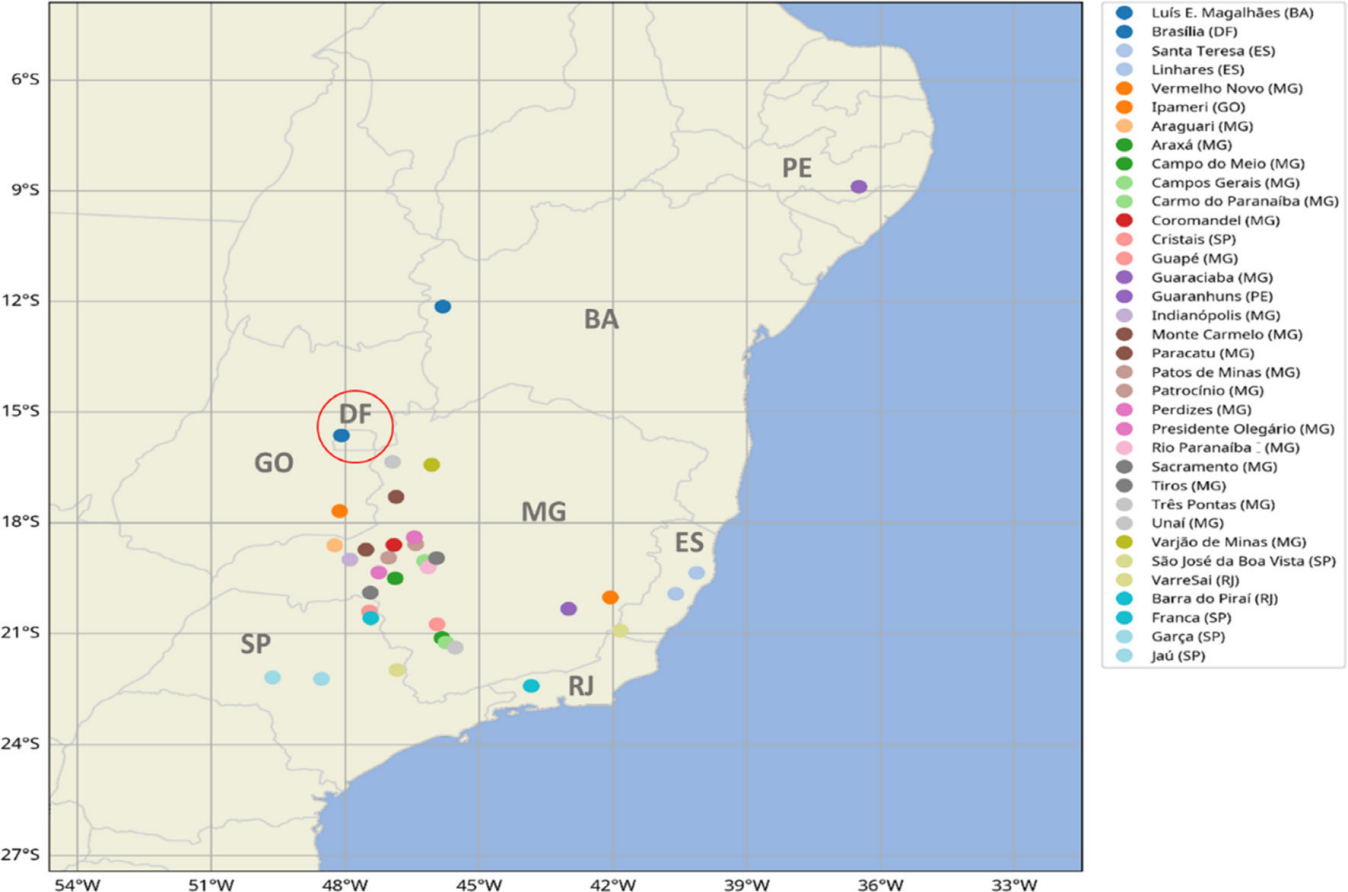
Collection sites of *L. coffeella* populations in Brazil.

*Leucoptera coffeella* populations were collected over a 2‐year sampling period (2011 to 2013). Thirty‐six coffee leaf miner populations were collected from commercial coffee plantations (*C. arabica* and *C. canephora*) in the Brazilian states of Minas Gerais (MG), Espírito Santo (ES), São Paulo (SP), Bahia (BA), Goias (GO), Distrito Federal (DF), Rio de Janeiro (RJ) and Pernambuco (PE) (Fig. 1). These states were chosen because of their importance in coffee production and history of intensive insecticide use. Fifteen hundred leaves with live *L. coffeella* larvae were selected from the middle third of randomly selected plants in commercial coffee plantations. These crops were geo‐referenced with a portable E‐trex Summit Hc GPS (Garmin, Olanthe, KS, USA) (Table 1). Leaves from each sampling site were placed in kraft paper bags (15‐kg capacity) and transported to the laboratory within 96 h.

**Table 1:** Location coordinates and information of sampling sites for field populations of *L. coffeella* in Brazilian states

Population	State	Coordinates	Temperature average (°C)	Rain (mm year^−1^)	Altitude (m)
1‐ Luís Eduardo Magalhães	BA	−12.14 S, −45.81 W	26.3	1452	720
2‐ Brasília	DF	−15.63 S, −48.09 W	25.4	1442	1172
3‐ Santa Teresa	ES	−19.93 S, −40.59 W	21.5	1162	174
4‐ Linhares	ES	−19.36 S, −40.13 W	24.3	1167	33
5‐ Vermelho Novo	MG	−20.03 S, −42.06 W	20.1	1335	640
6‐ Ipameri	GO	−17.69 S, −48.13 W	21.4	1468	764
7‐ Araguari	MG	−18.61 S, −48.24 W	25.8	1520	1065
8‐ Araxá	MG	−19.51 S, −46.88 W	23.6	1421	968
9‐ Campo do Meio	MG	−21.12 S, −45.83 W	21.1	1591	771
10‐ Campos Gerais	MG	−21.24 S, −45.74 W	20.5	1401	861
11‐ Carmo do Paranaíba	MG	−19.04 S, −46.22 W	21.2	1472	1120
12‐ Coromandel	MG	−18.60 S, −46.91 W	21.5	1765	921
13‐ Cristais	SP	−20.41 S, −47.46 W	21.1	1100	991
14‐ Guapé	MG	−20.75 S, −45.94 W	20.2	1400	765
15‐ Guaraciaba	MG	−20.34 S, −43.00 W	21.0	1611	1120
16‐ Garanhuns	PE	−08.89 S, −36.49 W	22.0	857	900
17‐ Indianópolis	MG	−19.00 S, −47.90 W	24.5	1460	800
18‐ Monte Carmelo	MG	−18.73 S, −47.54 W	23.7	1409	877
19‐ Paracatu	MG	−17.30 S, −46.86 W	26.4	1095	680
20‐ Patos de Minas	MG	−18.58 S, −46.42 W	25.9	1495	822
21‐ Patrocínio	MG	−18.95 S, −47.03 W	23.2	1477	970
22‐ Perdizes	MG	−19.35 S, −47.25 W	24.4	1510	1100
23‐ Presidente Olegário	MG	−18.39 S, −46.45 W	26.1	1485	945
24‐ Rio Paranaíba I	MG	−19.21 S, −46.14 W	21.3	1478	1071
25‐ Rio Paranaíba II	MG	−19.17 S, −46.09 W	21.3	1475	849
26‐ Sacramento	MG	−19.90 S, −47.44 W	20.4	1575	835
27‐ Tiros	MG	−18.96 S, −45.95 W	22.1	1485	1045
28‐ Três Pontas	MG	−21.38 S, −45.54 W	20.4	1502	900
29‐ Unaí	MG	−16.35 S, −46.94 W	26.3	1160	560
30‐ Varjão de Minas	MG	−16.43 S, −46.06 W	23.7	385	958
31‐ São José da Boa Vista	SP	−21.99 S, −46.84 W	21.4	1088	770
32‐ Varre‐Sai	RJ	−20.92 S, −41.84 W	24.1	298	678
33‐ Barra do Piraí	RJ	−22.42 S, −43.83 W	26.4	1572	362
34‐ Franca	SP	−20.58 S, −47.43 W	21.0	1644	578
35‐ Garça	SP	−22.19 S, −49.63 W	22.1	1465	681
36‐ Jaú	SP	−22.23 S, −48.53 W	23.5	1368	545

In the laboratory, leaves with undamaged mines (without openings or signs of parasitism or predation) were selected. The leaves were maintained in new wooden cages (55 × 60 × 90 cm) at 25 ± 1 °C, 70 ± 10% relative humidity (RH) under a 12 h:12 h, light:dark photoperiod until adult emergence. The adults from each population were then transferred to wooden cages (100 × 100 × 200 cm) containing coffee plant seedlings (Catuaí IAC‐144) as an oviposition substrate. Approximately 20 adults were placed per coffee seedling to maximize egg yield and obtain a large number of same‐aged caterpillars for the tests. The adult diet consisted exclusively of a 10% honey solution in deionized water, provided *ad libitum* through sterile cotton wicks fixed to the upper interior part of the cages. The cages were maintained in a glasshouse at 26 ± 1 °C and 70 ± 10% RH without insecticide applications.^32^ Following the introduction of adults into the cages, oviposition occurred, and larvae emerged 7 to 10 days after oviposition. The larvae used in the experiments were from the second generation, and within this generation, 3^rd^‐instar larvae aged 3–9 days were selected. To continuously acquire new eggs, the seedlings were removed from the cages and replaced with new ones every 3 days. A code name was assigned to each population for future identification.

### 2.3 Bioassays

#### 2.3.1 Control failure likelihood

We used the recommended label rates for each insecticide (Table 2).^24^ Thirty‐six field populations of coffee leaf miners from nine Brazilian states were used to determine control failure risk. Preliminary tests were done only with discs soaked in distilled water to observe larvae mortality after 48 h (<20% mortality in the control).^33^ The mortality of the control treatment in our study varied from 1.1% to 6.1%. The insects were considered dead when no reaction was detected after prodding with a fine‐tip brush. Filter paper discs (90‐mm diameter) were dipped in insecticide solutions that had been diluted in distilled water. The control treatment consisted of discs soaked in distilled water. These disks were fixed on a clothesline to dry in the shade and then placed individually in Petri dishes (9.0 cm diameter × 1.5 cm height). Ten 2^nd^‐instar *L. coffeella* caterpillars were placed on each plate using a fine point brush. The plates were then placed in an incubator chamber (model SP‐500) for 48 h at 25 ± 1 °C, 75% RH under a 12 h:12 h, light:dark photoperiod. The experiments were completely randomized with four replicates (10 larvae per replicate).

**Table 2:** Mortality (%) and control failure likelihood (%) of populations of *L. coffeella* using Brazilian recommended field doses

Population	State^a^	Insecticide^b^			
		Abamectin	Chorpyrifos	Deltamethrin	Prophenophos
1‐Luís Eduardo Magalhães	BA	48.3[39.6]*	37.4[51.6]*	47.8[40.3]*	38.6[51.6]*
2‐Brasília	DF	77.7[0.0]	48.8[39.0]*	55.5[30.6]*	61.2[23.5]*
3‐Santa Teresa	ES	80.7[0.00]	68.3[14.6]*	65.4[18.3]*	77.2[3.5]
4‐Linhares	ES	80.4[0.0]	87.1[0.0]	76.2[4.8]	77.1[3.6]
5‐Vermelho Novo	ES	88.7[0.0]	89.5[0.0]	77.6[3.0]	80.4[0.0]
6‐Ipameri	GO	84.5[0.0]	90.3[0.0]	75.2[6.0]	77.6[3.0]
7‐Araguari	MG	40.1[49.9]*	65.1[18.6]*	69.7[12.9]*	50.5[36.9]*
8‐Araxá	MG	68.9[13.9]*	66.3[17.1]*	65.4[18.3]*	77.8[2.8]
9‐Campo do Meio	MG	85.3[0.0]	70.7[11.6]*	80.9[0.0]	66.3[17.1]*
10‐Campos Gerais	MG	88.2[0.0]	86.3[0.0]	81.9[0.0]	78.6[1.8]
11‐Carmo do Paranaíba	MG	56.9[28.9]*	65.2[18.5]*	60.4[24.5]*	70.1[12.4]*
12‐Coromandel	MG	60.8[24.0]*	61.7[22.9]*	51.7[35.4]*	61.2[23.5]*
13‐Cristais	SP	84.2[0.0]	88.6[0.0]	90.1[0.0]	97.9[0.0]
14‐Guapé	MG	80.7[0.0]	78.6[1.8]	77.6[3.0]	78.6[1.8]
15‐Guaraciaba	MG	91.4[0.0]	94.7[0.0]	92.3[0.0]	80.7[0.0]
16‐Garanhuns	PE	31.3[55.8]*	90.5[0.0]	89.9[0.0]	87.8[0.0]
17‐Indianópolis	MG	55.1[31.1]*	50.5[36.9]*	56.8[29.0]*	53.4[33.3]*
18‐Monte Carmelo	MG	48.3[39.6]*	50.7[36.6]*	55.4[30.8]*	56.7[29.1]*
19‐Paracatu	MG	47.5[40.6]*	60.4[24.5]*	66.3[17.1]*	67.4[15.8]*
20‐Patos de Minas	MG	67.4[15.8]*	60.8[2.8]*	50.9[36.4]*	41.7[41.1]*
21‐Patrocínio	MG	64.1[19.9]*	60.3[24.6]*	40.7[47.4]*	61.9[22.6]*
22‐Perdizes	MG	81.2[0.0]	65.3[22.1]*	45.2[39.8]*	55.4[28.5]*
23‐Presidente Olegário	MG	87.4[0.0]	68.3[14.6]*	38.3[51.2]*	82.7[0.0]
24‐Rio Paranaíba I	MG	85.2[0.0]	50.4[37.0]*	61.3[23.4]*	57.6[28.0]*
25‐Rio Paranaíba II	MG	55.2[31.0]*	50.4[37.0]*	61.3[23.4]*	57.6[28.0]*
26‐Sacramento	MG	85.3[0.0]	44.5[40.5]*	87.9[0.0]	66.3[17.1]*
27‐Tiros	MG	87.4[0.0]	33.4[55.8]*	71.5[10.6]	70.6[11.8]*
28‐Três Pontas	MG	91.4[0.0]	92.3[0.0]	88.6[0.0]	84.2[0.0]
29‐Unaí	MG	96.7[0.0]	64.5[19.4]*	80.7[0.0]	44.9[41.6]*
30‐Varjão de Minas	MG	85.7[0.0]	46.8[41.5]*	69.7[12.9]*	60.0[25.0]*
31‐São José da Boa Vista	SP	78.3[2.1]	79.7[0.4]	62.2[22.3]*	78.3[2.1]
32‐VarreSai	RJ	88.5[0.0]	90.1[0.0]	87.2[0.0]	77.9[2.6]
33‐Rolim de Moura	RJ	74.3[7.1]	76.3[4.6]	80.7[0.0]	78.9[1.4]
34‐Franca	SP	72.9[17.5]*	45.0[44.8]*	88.4[0.0]	87.2[0.0]
35‐Garça	PR	90.5[0.0]	92.3[0.0]	91.1[0.0]	90.2[0.0]
36‐Jaú	SP	92.4[0.0]	81.8[0.0]	89.3[0.0]	84.6[0.0]
Locations with control failure		13	22	18	18

^a^ States indicate the Brazilian states where populations were collected.

^b^ Mortalities followed by an asterisk are significantly lower than the minimum efficacy threshold of 80% (one‐sided Z‐test at 95% confidence level with correction for continuity and Bonferroni correction; *n* = 120). Recommended label rate to abamectin (23.0 g a.i. L^−1^), chorpyrifos (1.8 g a.i. L^−1^), deltamethrin (0.3 g a.i. L^−1^) and prophenophos (1.1 g a.i. L^−1^).

Control failure likelihood (CFL) was estimated from the mortality rates observed after label‐rate doses of each insecticide were applied to coffee leaf miners (Table 2).^24^ CFL was calculated as 100 − [observed mortality × 100] ÷ expected mortality (80%).^10^ Mortality <80% indicates failure of control. Negative CFL values indicate negligible risk of control failure.

#### 2.3.2 Resistance

Resistance in nine Brazilian states was evaluated using the same experimental units described for the control failure bioassay. Three different concentrations of insecticides were tested in the preliminary tests and a total of 13 concentrations were used in the definitive bioassays. Stock solutions of the insecticides were prepared in 100 mL distilled water. Serial dilutions of 800, 400, 200, 100, 10, 5, 1, 0.5, 0.2, 0.05, 0.02, 0.01 and 0.005 g a.i. L^−1^ were prepared from each stock solution based on the registered label concentrations. A control treatment of distilled water was included for each tested insecticide.

#### 2.3.3 Enzymatic activity

The selection of populations for enzymatic assays was based on a stratified sampling approach designed to represent the complete continuum of insecticide resistance pressures identified in the preliminary bioassays of control failure likelihood and resistance with 36 field populations of *L. coffeella*.

Three distinct profiles were established based on control failure likelihood and resistance ratios: (i) high selection pressure: populations showing control failure for three or more insecticides with median resistance ratio (RR_50_) > 1000; (ii) moderate selection pressure: populations with control failure for one to two insecticides and RR_50_ between 50 and 500; and (iii) low infestation pressure: populations maintaining field control (no control failure) despite detectable resistance mechanisms.

The Cerrado Mineiro populations (Rio Paranaíba I, Rio Paranaíba II and Carmo do Paranaíba) were selected as high selection pressure representatives based on their consistent control failure for three to four insecticides and the presence of extremely high resistance ratios within the group, with RR_50_ values reaching 1816 and 2812 in some populations for specific insecticides. Their selection also was supported by their location in a region characterized by environmental conditions that favor rapid pest development cycles (≤11 days in favorable seasons) and intensive coffee production systems.^21^ Franca was chosen as the moderate selection pressure representative, demonstrating control failure for two insecticides with intermediate resistance levels (RR_50_ = 95.1 for chlorpyrifos). Santa Teresa and Garanhuns were selected as low infestation pressure representatives, maintaining effective field control for most insecticides despite specific high resistance patterns (RR_50_ > 26 000 for deltamethrin and abamectin), indicating reduced current selection pressure. This stratified selection ensures that the enzymatic activity analysis captures the biochemical diversity across the complete resistance spectrum observed in Brazilian coffee‐growing regions. Absorbance was determined using a spectrophotometer (SP‐220; Biospectro, Curitiba, Brazil). All experiments were conducted with three replicates and in triplicate.

Three replicates were used for each enzyme assay and insect population. For each repetition, sixty *L. coffeella* caterpillars were removed from leaves in the field with active mines, placed in 14‐mL tubes, and kept at –20 °C until required for the assays. To determine AChE and GST activity, 30 caterpillars were placed in 3 mL of 0.1 m phosphate buffer (pH 7.5) containing 0.3% triton X‐100. The remaining 30 caterpillars were placed in 3 mL 0.05 m glycine‐sodium hydroxide (NaOH) buffer (pH 8.0) to determine PhTE activity. Enzyme extracts were obtained by macerating cellular material with the buffer solution specific for each enzyme, at 4 °C, and using a porcelain mortar and pestle. After maceration, the solution was transferred to 2‐mL microtubes with caps and centrifuged at 10.600 × *g*, for 5 min for PhTE analysis, and 15 min for the AChE and GST assays. The supernatant was then removed and kept on ice until the protein concentration and enzymatic activity was determined.

The protein concentrations in the *L. coffeella* enzyme extracts were determined using BSA (0.2 mg mL^−1^) as a protein standard.^34^ AChE activity was evaluated by measuring the product of an enzymatic reaction with the ACT substrate, using a modified version of the process described in Ellman *et al*.^35^ The reaction mixture contained 500 μL 0.1 m phosphate buffer (pH 7.5), 100 μL 1 mm ATC, 300 μL 0.1 mm DTNB and 100 μL enzyme. This mixture was incubated at room temperature (RT). All treated larvae were kept at 26 °C under a photoperiod of 16 h:8 h, light:dark, and AChE activity was determined by measuring absorbance 405 nm (measured in spectrophotometer) every 30 s for 7 min. GST activity was measured using 1000 μL 15 mm glutathione and 20 μL 150 mm DCNB substrate. The reagents were homogenized and kept for 3 min at RT. Then, 250 μL enzymatic extract was added to the test tube, and the entire contents were transferred to a cuvette where absorbance was read at wavelength 340 nm (measured in spectrophotometer) at intervals of 30 s for 5 min. PhTE activity was determined using paraoxon as a substrate at 405 nm, based on Guedes *et al*.^36^ with some modifications. The reaction mixture contained 700 μL 0.05 m NaOH‐glycine buffer (pH 8.0), 500 μL 3 mm paraoxon and 100 μL enzyme. Each assay was performed with three replicates and a corresponding control containing 60 mm EDTA to inactivate PhTE activity. The reaction mixtures were incubated for 24 h at 37 °C.^36^

### 2.4 Data analysis

The data from the resistance monitoring and control failure likelihood surveys were subjected to a unilateral Z‐test at the 95% confidence level and corrected for continuity.^37^ The Bonferroni correction was used to correct the *P*‐values. The concentration–mortality bioassay data from each population were corrected for the number of dead insects in the control (i.e. natural mortality).^38^ The insecticide mortalities of the different insect populations were compared by year via probit analysis using the PROCPROBIT procedure in SAS.^39^ The RR_50_ was calculated as the ratio of the lethal concentration (LC_50_) of each population by the LC_50_ of the susceptible population. The 95% confidence intervals for the resistance ratios were estimated according to Robertson *et al*.^40^ and considered significant if not including the value 1. Statistically significant differences (*P* < 0.05) in *L. coffeella* enzyme activity were analyzed using one‐way ANOVA and Tukey's honestly significant difference (HSD) test.

## 3 RESULTS

**Figure 2:**
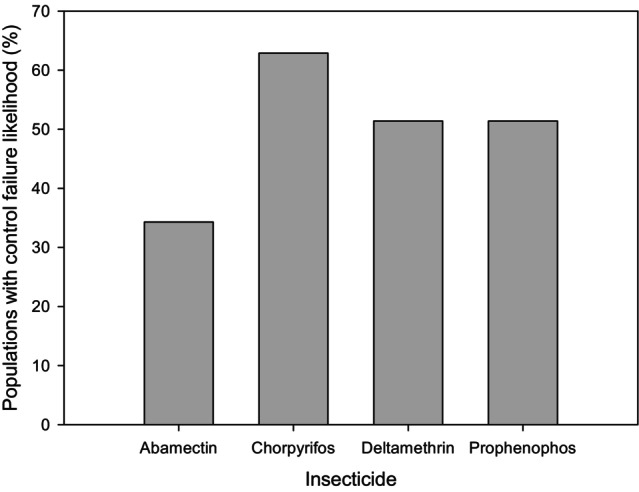
Percentage of populations with control failure likelihood of the *L. coffeella* using Brazilian recommended field doses of abamectin, chorpyrifos, deltamethrin and prophenophos.

Abamectin, chlorpyrifos, deltamethrin and profenophos exhibited high control failure risks at 34.3%, 62.9%, 51.4% and 51.4% of the coffee leaf miner populations, respectively. Chlorpyrifos, deltamethrin and profenophos showed low efficacy in more than half the populations (Table 2; Fig. 2).

The RR_50_ values showed that the coffee leaf miner populations exhibited low to very high levels of resistance to abamectin (175‐ to 26 478‐fold), chlorpyrifos (44.2‐ to 1816‐fold), deltamethrin (45.2‐ to 27 603‐fold) and profenophos (11.1‐ to 33.2‐fold) (Table 3). Resistance levels were classified according to the RR_50_ values as follows: low (<100‐fold), moderate (100–500‐fold), high (501–5000‐fold) and very high (>5000‐fold).^40^ The populations with the highest RR_50_ values (very high resistance) were from Santa Teresa (deltamethrin: 27603), Garanhuns (abamectin: 26478) and Rio Paranaiba II (chlorpyrifos: 1816) (Table 3).

**Table 3:** Relative toxicity of neurotoxic insecticides to 3^rd^‐instar larvae from Brazilian populations of *L. coffeella*

Insecticide	Population	State	*n*	Slope ± SE	LC_50_ g a.i. L^−1^ (95% CI)	*χ* ^2^ (df)	*P*	RR_50_
Abamectin	Rio Paranaíba I	MG	400	1.07 ± 0.012	5.30(3.80–7.20)	7.20(8)	0.12	175.00(161.00–194.00)*
	Rio Paranaíba II	MG	360	1.00 ± 0.007	0.03(0.02–0.04)	5.97(7)	0.31	1.00(0.52–2.11)
	Carmo Paranaíba	MG	360	1.23 ± 0.001	84.40(69.10–103.00)	5.73(7)	0.33	2812.00(2801.00–2836.00)*
	Franca	SP	400	0.82 ± 0.000	25.40(16.00–36.20)	2.55(8)	0.53	846.00(827.00–858.00)*
	Santa Teresa	ES	400	2.70 ± 0.001	403.00(351.00–451.00)	5.10(8)	0.16	13 435.00(13 411.00–13 467.00)*
	Garanhuns	PE	400	1.03 ± 0.001	794.00(612.00–1048.00)	2.32(8)	0.51	26 478.00(26 468.00–26 489.00)*
Chlorpyrifos	Rio Paranaíba I	MG	400	0.54 ± 0.000	17.80(8.00–33.00)	0.24(8)	0.99	44.20(37.10–50.40)*
	Rio Paranaíba II	MG	400	1.54 ± 0.001	727.00(600.00–878.00)	8.46(8)	0.08	1816.00(1805.00–1826.00)*
	Carmo do Paranaíba	MG	400	0.28 ± 0.000	0.40(0.005–6.33)	5.76(8)	0.33	1.00(0.11–2.95)
	Franca	SP	400	0.62 ± 0.001	38.00(6.90–77.30)	2.61(8)	0.54	95.10(90.20–100.80)*
	Santa Teresa	ES	400	0.63 ± 0.002	67.80(40.00–107.00)	9.13(8)	0.06	169.50(151.00–176.00)*
	Garanhuns	PE	400	0.44 ± 0.002	142.00(67.30–259.40)	1.34(8)	0.72	355.80(341.00–374.00)*
Deltamethrin	Rio Paranaíba I	MG	320	0.88 ± 0.001	3.70(2.70–4.80)	12.84(6)	0.08	60.80(50.70–73.40)*
	Rio Paranaíba II	MG	320	0.71 ± 0.001	2.70(1.20–4.71)	2.59(6)	0.77	45.20(39.70–51.90)*
	Carmo do Paranaíba	MG	360	1.13 ± 0.003	33.30(24.61–45.20)	4.56(7)	0.10	555.00(531.00–579.00)*
	Franca	SP	360	0.44 ± 0.001	0.061(0.007–0.80)	1.44(7)	0.70	1.00(0.22–1.89)
	Santa Teresa	ES	360	0.61 ± 0.002	1656.00(931.00–4540.00)	5.83(7)	0.05	27 603.00(27 569.00–27 644.00)*
	Garanhuns	PE	400	0.85 ± 0.018	1281.00(900.00–3251.00)	6.86(8)	0.09	21 357.00(21 311.00–21 377.00)*
Prophenophos	Rio Paranaíba I	MG	400	1.79 ± 0.002	41.20(34.80–49.00)	5.01(8)	0.17	13.90(3.11–20.50)*
	Rio Paranaíba II	MG	400	0.95 ± 0.001	91.00(65.40–136.90)	2.52(8)	0.53	30.80(15.20–42.60)*
	Carmo do Paranaíba	MG	400	0.46 ± 0.001	3.00(0.60–7.40)	4.08(8)	0.25	1.00(0.11–3.12)
	Franca	SP	360	1.85 ± 0.003	49.20(34.10–50.70)	5.02(7)	0.20	16.60(10.40–27.60)*
	Santa Teresa	ES	360	0.32 ± 0.001	98.10(65.00–131.30)	4.55(7)	0.65	33.20(20.70–39.70)*
	Garanhuns	PE	360	0.12 ± 0.000	11.10(10.30–15.50)	3.14(7)	0.74	11.10(1.41–20.70)*

*Note*: *in the RR_50_ indicates a significant difference from the standard susceptible population suggested by Robertson *et al*.^40^

Abbreviations: *n*, total number of insects per bioassay. χ^2^, calculated chi‐squared; df, degrees of freedom; CI, 95% confidence interval; LC_50_, lethal concentration to kill 50% of the population; RR_50_, resistance ratio, obtained dividing the LC_50_ of each population by the LC_50_ of the susceptible; SEM, standard error of the mean.

The AChE activity varied among populations, with the lowest activity observed in Rio Paranaíba II (5.68 ± 0.59 mm min^−1^ mg^−1^ protein), whereas the highest activities were recorded in Franca (13.26 ± 2.09), Carmo do Paranaíba (12.48 ± 9.59), Rio Paranaíba I (11.85 ± 2.23), Garanhuns (10.12 ± 1.32) and Santa Teresa (12.03 ± 1.01). The GST activity differed among some populations. Rio Paranaíba I exhibited the highest activity (0.10 ± 0.09 mm min^−1^ mg^−1^ protein), followed by Carmo do Paranaíba (0.06 ± 0.02). Populations from Santa Teresa (0.03 ± 0.02), Garanhuns (0.02 ± 0.01) and Rio Paranaíba II (0.02 ± 0.02), as well as Franca (0.02 ± 0.00), showed similar activities, which were the lowest observed. The PhTE activity was generally low across all populations, ranging from 2.03 × 10^−5^ ± 1.17 × 10^−6^ μmol h^−1^ mg^−1^ protein in Rio Paranaíba I to 3.47 × 10^−5^ ± 3.09 × 10^−7^ in Franca, with no significant differences observed among populations (Table 4).

**Table 4:** Specific activity of acetylcholinesterase (AChE), glutathione *S*‐transferase (GST) and phosphotriesterase (PhTE) in larvae of Brazilian populations of *L. coffeella*

Populations	State	AChE*	GST*	PhTE*
		mm min^−1^ mg^−1^ protein	mm min^−1^ mg^−1^ protein	μmol h^−1^ mg^−1^ protein
Rio Paranaíba II	MG	5.679 ± 0.59b	0.021 ± 0.02c	2.113 × 10^−5^ ± 1.35 × 10^−6^a
Carmo do Paranaíba	MG	12.477 ± 9.59a	0.057 ± 0.02b	2.171 × 10^−5^ ± 1.08 × 10^−6^a
Franca	SP	13.260 ± 2.09a	0.015 ± 0.00c	3.470 × 10^−5^ ± 3.09 × 10^−7^a
Rio Paranaíba I	MG	11.846 ± 2.23a	0.097 ± 0.09a	2.026 × 10^−5^ ± 1.17 × 10^−6^a
Garanhuns	PE	10.122 ± 1.32a	0.018 ± 0.01c	2.154 × 10^−5^ ± 1.11 × 10^−6^a
Santa Teresa	ES	12.031 ± 1.01a	0.026 ± 0.02c	2.744 × 10^−5^ ± 1.00 × 10^−6^a

* Means followed by the same letter in the column did not differ statistically by Tukey's HSD test at *P* < 0.05.

## 4 DISCUSSION

### 4.1 Control failure

We exposed 36 *L. coffeella* populations to abamectin, chlorpyrifos, deltamethrin and profenophos to determine control failure likelihood (CFL), and found risk of control failures in more than half of these Brazilian *L. coffeella* populations (Table 2; Fig. 2). Coffee leaf miner control failure occurs when the efficacy of a previously effective insecticide becomes less efficacious (mortality <80%), and it is usually associated with resistance.^25^ In Brazil, significant control failures have been observed for insecticides, including organophosphates.^30^ Several groups, including more recent insecticides such as diamides, already show moderate resistance records.^25^ As mentioned previously, chemical control remains the main management strategy in Brazilian coffee plantations.^26^ The insecticides evaluated in this study were selected based on their common use in coffee pest management. Siqueira *et al*.^41^ observed 100% control failure of abamectin in Brazilian populations of *Tuta absoluta* (Meyrick) (Lepidoptera: Gelechiidae). Torres *et al*.^42^ showed failures in organophosphate control in populations of *Anthonomus grandis grandis* (Boheman) (Coleoptera: Curculionidae). Rocha *et al*.^30^ showed that chlorpyrifos failed to control populations of *L. coffeella* in Minas Gerais (Brazil) and Silva *et al*.^43^ found deltamethrin control failures associated with triazophos in Brazilian populations of *T. absoluta*.

### 4.2 Resistance and enzymes

The selected six *L. coffeela* to study resistance and enzymatic activity showed different degrees of resistance to the four evaluated insecticides. Resistance levels in the different source regions ranged from susceptible to very high, with profenophos showing low resistance. However, abamectin, chlorpyrifos and deltamethrin showed high resistance levels in most of the populations. Populations of *L. coffeela* showed varying resistance levels to insecticides from the same chemical group. For example, the Rio Paranaíba population showed low resistance to profenophos and high resistance to chlorpyrifos, as was reported for *L. coffeell*a with organophosphates.^27^, ^28^, ^29^ These facts contributed to higher and lower resistance levels in certain regions and suggest that resistance management should go beyond simply rotating insecticides with different modes‐of‐action.

Differences in resistance levels between neighboring regions, such as Rio Paranaíba II and Rio Paranaíba I (±15 km), indicate that collection distance is not associated with resistance,^28^ but sample size may influence the detectability of resistance.^44^ This was demonstrated by Shah *et al*.^44^ who found that different sample sizes can cause under or overestimation of resistance levels within the same region. The type of resistance associated with organophosphate, pyrethroid and avermectin insecticides also was not identified for different populations of *Alabama argillacea* (Hübner) (Lepidoptera: Noctuidae).^43^

A particularly notable observation was made for the Rio Paranaíba I population, which consistently exhibited the highest activities of AChE and GST, in addition to showing resistance to all tested insecticides (Tables 3 and 4). However, this relationship was not linear across populations, as some with lower enzymatic activity displayed moderate resistance or even susceptibility, particularly to chlorpyrifos (Table 3). This pattern suggests that, although detoxifying enzymes play a significant role in resistance, their isolated expression does not fully account for the observed phenotypic differences, as documented in *T. absoluta*, where not all resistant populations exhibited uniformly elevated levels of resistance‐associated enzymes, including cytochrome P450‐dependent monooxygenases, carboxyl/choline esterase (CCE) and GST.^45^ It is therefore likely that multiple mechanisms, including metabolic resistance and target‐site alterations, act simultaneously, with the magnitude of the response dependent on the genetic composition of each population. Previous studies indicate that *L. coffeella* exhibits high genetic variability,^46^, ^47^ which is reflected in the pronounced variation in insecticide susceptibility or resistance.^25^, ^30^ This perspective has been recently reinforced by the availability of the reference genome of *L. coffeella*, which revealed 16407 bp circular mithocondrial g, providing a foundation for future investigations into molecular resistance mechanisms.^48^ These findings support the hypothesis that intraspecific differences may result in nonlinear associations between enzymatic activity and resistance. Consequently, the genetic diversity of this species may contribute to the absence of a predictable biochemical pattern, highlighting the need for integrating genetic and biochemical analyses in future resistance monitoring efforts.

Reductions in insect sensitivity to insecticides can be caused by different mechanisms including metabolic changes. Metabolic resistance can occur directly, by increasing the metabolism of insecticides and their products or by sequestration of toxic compounds, or indirectly by protecting against oxidative stress induced by insecticide exposure. Biochemical resistance to some insecticides, such as organophosphates, organochlorines and pyrethroids, has been mainly associated with the detoxification of these compounds by increased activity of GST, esterases and cytochrome P450‐dependent monooxygenases.^48^ GST conjugates reduce glutathione from these insecticides or the toxic products of their metabolism. Greater GST activity, resulting from gene amplification or increased transcription has been directly related to resistance.

Resistance in *Helicoverpa assulta* (Guenée) (Lepidoptera: Noctuidae) to indoxacarb was attributed to a significant increase in carboxylesterase and GST activity.^49^ The dose‐dependent upregulation of GST gene expression and enzyme activity were observed when *Helicoverpa armigera* (Hübner) (Lepidoptera: Noctuidae) larvae were exposed to a blend of organophosphate (chlorpyrifos and dichlorvos) and pyrethroid (cypermethrin) pesticides.^16^ This upregulation could be responsible for larval tolerance and adaptation at rates as high as 15 ppm, as indicated by larval survival.^50^ The same study reported significantly higher GST activity in the crude homogenate from the midguts of *Bombyx mori* L. (Lepidoptera: Bombycidae) after long‐term exposure to phoxim and fenpropathrin, and found elevated expression of the epsilon‐class GST gene (BmGSTe2) in the midguts of phoxim‐ and fenpropathrin‐selected insects.

Acetylcholinesterase activity also is involved in insect resistance to insecticides. This essential enzyme terminates synaptic transmission in the cholinergic nervous system of most animals, including insects, by hydrolyzing the neurotransmitter acetylcholine to choline and acetate at cholinergic synapses and neuromuscular junctions. Owing to its crucial role, AChE is the target of insecticides such as organophosphate and carbamate that associate with and inhibit AChE activity.^51^ However, reduced AChE sensitivity has led to the development of resistant populations.

*Nauphoeta cinerea* (Olivier) (Blattaria: Blaberidae) showed a marked decrease in AChE activity and antioxidant status, with a concomitantly significant elevation in lipid peroxidation levels,^52^ after exposure to chlorpyrifos. Chlorpyrifos, at all studied concentrations, significantly reduced AChE activity in *Spodoptera exigua* (Hubner 1808) (Lepidoptera: Noctuidae).^19^ The insecticidal compounds stigmasterol and 1‐hexacosanol inhibited AChE activity in *Culex quinquefasciatus* (Say) (Diptera: Culicidae) and *Aedes aegypti* L. (Diptera: Culicidae).^53^ Molecular studies indicate that point mutations of arthropod neuronal AChE confer insensitivity to AChE. Additionally, several cases of AChE gene duplication have been related to the adaptation mechanism that compensates for the possible fitness cost caused by mutated AChE.^51^

The widespread and variable insecticide resistance observed in *L. coffeella* populations highlights the urgent need for continuous monitoring of insect population responses to insecticides. This monitoring is essential to identify fields where changes in control strategies must be made, as the frequent use of the same mode‐of‐action insecticide induces the development of resistance and ensures insect survival. Outbreaks caused by surviving insects may encourage farmers to increase the amounts of insecticide, leading to repetitive use with higher health risks and even more resistance developing. Therefore, our findings emphasize that resistance management must be integrated into broader integrated pest management (IPM) strategies, which depend on the identification of natural enemies, application of economic threshold levels, reduction of selection pressure and management of agronomic variables. Several factors, such as short generation times, high fecundity, high exposure to extensively used insecticides, and hot and dry weather, contribute to the rapid selection of insecticide resistance in *L. coffeella*. Furthermore, the present study created avenues for future research on the genetics of molecular resistance in *L. coffeella* populations.

This study provides a robust analysis of AChE, GST and PhTE activities across Brazilian *L. coffeella* populations, yet future research should incorporate cytochrome P450 monooxygenases and carboxylesterases to achieve a comprehensive metabolic resistance profile. As highlighted by recent reviews,^54^ these enzyme systems play pivotal roles in resistance to key insecticide classes including pyrethroids and abamectin. Our findings on the spatial variation of established resistance mechanisms create a critical foundation for such subsequent work, enabling targeted investigation of P450 and esterase contributions within populations already characterized for AChE, GST and PhTE activities.

## 5 CONCLUSIONS

Abamectin, chlorpyrifos, deltamethrin and profenophos exhibit significant control failure risks in *L. coffeella* populations across Brazilian coffee‐growing regions. Resistance levels ranged from low to extremely high, with certain populations demonstrating exceptionally elevated resistance, particularly to deltamethrin, abamectin and chlorpyrifos. Altered activities of AChE and GST enzymes were observed, confirming the presence of biochemical resistance mechanisms. These findings highlight widespread insecticide resistance that compromises chemical control efficacy, underscoring the urgent need for resistance monitoring and IPM strategies to sustain coffee production.

## CONFLICT OF INTEREST

No professional competing interests exist.

## Data Availability

The data that support the findings of this study are available from the corresponding author upon reasonable request.
